# Simple Fixed Functional Space Maintainer

**DOI:** 10.5005/jp-journals-10005-1272

**Published:** 2015-02-09

**Authors:** Puneet Goenka, Aditi Sarawgi, Nikhil Marwah, Parvind Gumber, Samir Dutta

**Affiliations:** Reader, Department of Pediatric and Preventive Dentistry, Mahatma Gandhi Dental College, Jaipur, Rajasthan, India; Postgraduate Student, Department of Prosthodontics, Krishnadevaraya College of Dental Sciences and Hospital, Bengaluru, Karnataka, India; Associate Professor, Department of Pedodontics, Mahatma Gandhi Dental College Jaipur, Rajasthan, India; Senior Lecturer, Department of Oral Pathology, Mahatma Gandhi Dental College and Hospital, Jaipur, Rajasthan, India; Senior Profesor, Department of Pedodontics, Government Dental College Jaipur, Rajasthan, India

**Keywords:** Space maintainer, Fiber-reinforced composite, Esthetic.

## Abstract

Premature loss of a primary tooth is one of the most common etiology for malocclusion. Space maintainers are employed to prevent this complication. In anterior region, esthetics is an important concern along with function and space management. Fiber-reinforced composite (FRC) retained space maintainer solves all these purposes ef ficiently and ef fectively. In addition, the technique is simple and the appliance is very comfortable inside the oral cavity. Here is a case of premature loss of anterior primary tooth which was replaced by FRC retained esthetic functional space maintainer. The appliance was found to be functioning satisfactorily inside the oral cavity till the last visit (1 Year).

**How to cite this article:** Goenka P, Sarawgi A, Marwah N, Gumber P, Dutta S. Simple Fixed Functional Space Maintainer. Int J Clin Pediatr Dent 2014;7(3):225-228.

## INTRODUCTION

Tooth material arch length discrepancy is the most common causes for malocclusion. An excess arch length or space as compared to tooth material results in spacing in the dentition. On the other hand, when there is arch length deficiency (relative to the tooth material), crowding results. Premature loss of primary tooth is one of the etiologies for the loss of space or arch length. When a primary tooth is lost prematurely the teeth adjacent to the created space tend to drift into the space resulting in the loss of space required for the proper alignment of the succedaneous teeth in the arch, thereby resulting in crowding and other types of malocclusion.

In the situation where an anterior primary tooth is lost before schedule, the drifting of adjacent teeth into the space rarely occurs, thus making space management a minimal concern to the pedodontist. In contrast, the premature loss of an anterior tooth definitely results in an unesthetic smile and difficulty in biting, i.e. loss of function thus making the situation which cannot be left unattended. The primary anteriors are lost most commonly at the age of 2 to 4 years, because of trauma or caries. This is the age, when the child starts moving out of his house and he or she starts indulging in various outdoor and sports activities. At this age, the child also starts attending the play school and thus the peer group starts playing its role. Loss of an anterior tooth at this age, apart from the functional problems associated with it, may result in psychological trauma to the child. The problem may become serious and deep seated and may result in an imbalanced emotional development of the child. Along with this the teeth adjacent to the edentulous space may drift into the space, although to a lesser extent increasing the potential for developing malocclusion at a later stage.

An assortment of appliance has been developed by various investigators to deal with space management in case of early loss of a primary tooth. The choice can be between a removable and a fixed one; or it can be unilateral or bilateral; and functional or nonfunctional. The selection of the appliance depends upon a number of factors^1^ including the child's stage of dental development, the dental arch involved, the tooth missing and the status of the teeth adjacent to the lost tooth.

Over the last few years, the development of fiber-rein-forced composites (FRC) has offered the dental profession with the possibility of fabricating adhesive, esthetic, and metal-free tooth replacements even in the case of molar teeth.^2^ Structurally, the FRC is made up of two components: the fibers and the resin matrix. The resin matrix serves as a carrier, protector, and load-splicing medium around the fibers. To improve the mechanical properties of composite resins and to optimize the mechanical behavior of the material, specifically-oriented fller materials, such as glass fibers, aramid fibers, carbon/graphite fibers, and ultra high molecular weight polyethylene (UHM-WPE) fibers, have been proposed.^3^

This case report explains the method of fabrication of a fixed esthetic functional space maintainer using fiber reinforced composite resin, which was used to replace a prematurely lost primary central incisor. The article also compares the technique employed with other techniques available to replace a lost anterior primary tooth.

**Fig. 1:**
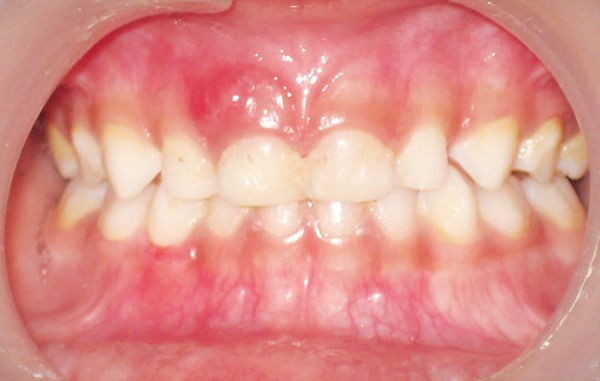
Affected right maxillary first deciduous central incisor

**Fig. 2:**
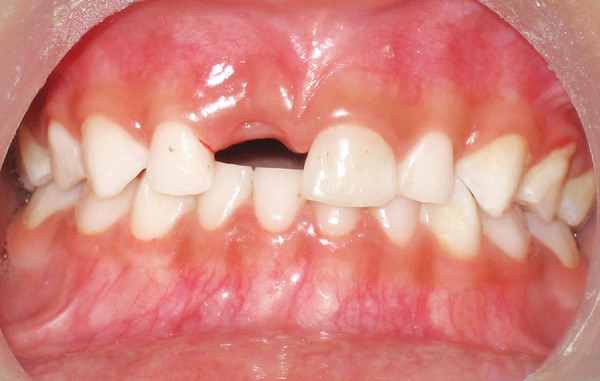
Healed extraction socket

**Fig. 3:**
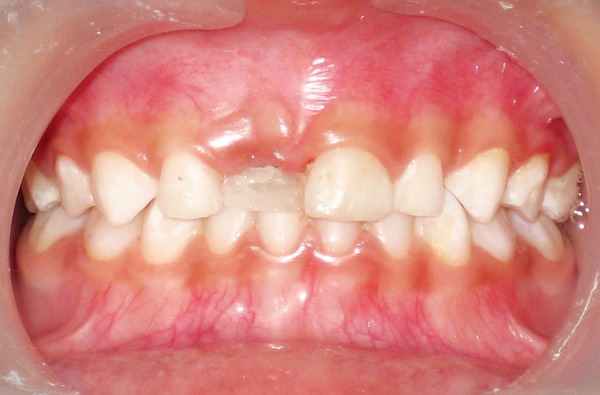
Bonding of fiber-reinforced composite

### Technique

A 4-year-old girl reported to the dental clinic with history of pain in the upper anterior tooth for 2 days. The pain was of severe and continuous type. The parents reported a bicycle fall at the age of three, i.e. a year back. The tooth was sore at that time for a few days but symptoms subsided thereafter. Parents did not report to any dentists at the time of trauma.

On clinical examination it was found that the maxillary right primary central incisor was mobile (grade III) and was tender on palpation. Soft tissue examination revealed that the gingiva in relation to the affected tooth was red and swollen suggesting the presence of a peri-apical abscess in relation to the tooth. No draining sinus was present in association with it (Fig. 1).

Radiograph showed considerable bone loss around the maxillary right primary central incisor. Extraction of the tooth followed by a fixed esthetic functional space maintainer was decided as the treatment plan for the tooth. The tooth was extracted under antibiotic coverage. The procedure was carried out under local anesthesia and the tooth was extracted atraumatically. The patient was kept on weekly recall.

After 2 weeks, the socket was found to have healed uneventfully (Fig. 2). A strip of FRC resin was cut approximately of the length equal to the distance from the distal surface of the left upper central incisor to the distal surface of the right upper lateral incisor. The palatal surfaces of the teeth present on both the sides of the extraction space were conditioned with 37% phosphoric acid. Bonding agent was applied and was cured as per manufacturer's instructions. A thin layer of fowable composite was applied over the etched surfaces of the abutment teeth and following this the FRC strip was adapted over the palatal surface extending from the distal surface of the maxillary right primary lateral incisor through the distal surface of the maxillary left primary central incisor. Then the strip placed in position was cured using a light curing unit for sufficient duration. The occlusion was checked for any premature contact (Fig. 3).

**Fig. 4:**
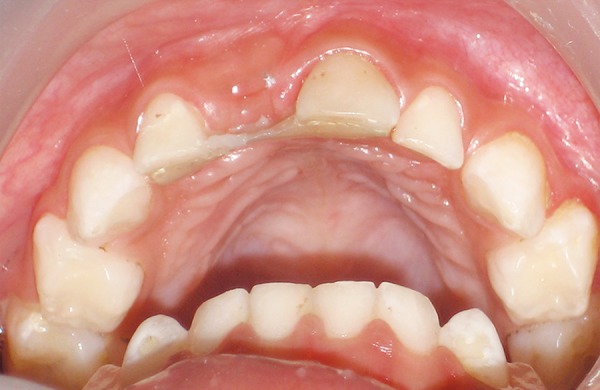
Fiber-reinforced composite in place

Now the tooth was prepared with the help of composite resin. For this, composite resin of appropriate shade was used in small increments and each of them was light cured for sufficient time. The complete tooth was prepared through free hand build up and then fnishing and polishing of the tooth was done so as to achieve morphological resemblance to the natural tooth. Occlusion was again checked to correct any pre-mature contact if present (Fig. 4).

**Fig. 5:**
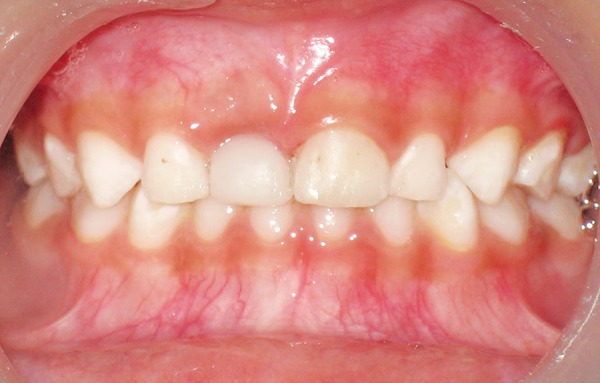
The fnal appliance

The patient was recalled after 1 week to check the integrity of the appliance in the mouth. The appliance was found to be satisfactory both functionally as well as esthetically (Fig. 5). The patient was kept on 3 months regular recall visits and the appliance was found to be functioning well inside the oral cavity till the last visit (9 months).

## DISCUSSION

Premature loss of a primary tooth is one of the common causes for malocclusion in permanent dentition. This is mainly because the teeth adjacent to the space created by the loss of the deciduous tooth, tend to drift into the space resulting in tooth material arch length discrepancy thereby predisposing the dentition to malocclusion. To combat this problem a number of space maintainers have been designed which can either be a removable or a fixed; functional or nonfunctional and unilateral or bilateral. The selection can be tailored depending upon the need of the situation.

In a situation where an anterior tooth is prematurely lost, a removable functional space maintainer is the one most commonly employed. Unfortunately, there are a number of drawbacks associated with the use of a removable space maintainer, the most important among them being, dependency on the patient (child) for the success of the appliance. This is because the child may not wear it or may wear at his or her convenience. In other words, the efficiency and effectiveness of a removable space main-tainer more or less depends on the patient's compliance than on the design and quality of the appliance. Other problems include accidental ingestion or aspiration of the appliance, breakage of the appliance during function or when it is out of mouth and loss of the appliance. In addition to this, a removable appliance may have a deleterious effect on the involved soft tissue and periodontium. A fixed appliance like the one used in this case is almost free from all such shortcomings.

Fixed esthetic space maintainers to replace a prematurely lost anterior tooth have also been tried earlier. The most commonly used technique involves the adaptation of stainless steel bands on the second deciduous molars and a wire soldered to them joining the two bands. The wire was adapted such that it runs close to the palatal surfaces of all teeth anterior to the teeth banded. An additional wire attachment was soldered to this wire which extends over the edentulous space and holds the pontic in place. The problem with this appliance is its excessive fexibility and lack of support. In adittion, the technique is very complicated and time consuming.

The technique employed in the case presented here makes the use of FRC resin for the fabrication of a fixed esthetic functional space maintainer. Fiber reinforced composite resin is relatively new to pediatric dentistry, but has been extensively used in removable prostho-dontics, fixed partial dentures, periodontal splints, and in orthodontic treatment as a retention splint.^1^ Fiber reinforced composite resin have also been used in the past to replace missing teeth.^4^ In pediatric dentistry they have been tried in the fabrication of space main-tainers for the posterior segment with limited success. Kargul et al^5^ in a clinical trial concluded that the glass fiber reinforced composite resin (GFRCR) space main-tainers, used in the case of missing one or more primary molars functioned well during a short period. Similar results were also confirmed in another study done by Kirzioğlu and Ertürk.^6^ The investigators also added that the clinical advantages of the GFRCR space maintainers were that they: provided cost and time savings; did not require a cast model; did not require a second visit; were easy to apply; provided reliable adhesive bonding; provided long-term retention; were used when there was an indication of metal allergy; were easy to clean; had a natural feel and were esthetic. Moreover they do not make any contact with adjacent periodontal tissues, thereby eliminating periodontal problems associated with conventional fixed space maintainers.^5,7,8^ As compared to the conventional fixed space maintainers (using ba nds a nd wi res) t he applia nce i s more est het ic as it do es not make use of metal. McDonald and Avery suggested that the band and loops should be removed once a year to inspect, clean, and apply fuoride to the tooth. Fiber-reinforced composite resin eliminates these annual steps.^9^

In the present case, a free hand build up was done using light cure composite resin to replace the lost primary central incisor. The natural crown of the extracted tooth can also be used as the pontic if it is not damaged by caries or trauma. When the extracted tooth crown is used there is an added advantage of preservation of natural tooth form, contour and translucency. Immediate replacement of an extracted tooth using FRC with the natural tooth as the pontic has been successfully tried by a number of investigators.^10-12^

Wijlen PV reported two cases in which a missing permanent tooth was replaced using FRCR and a free hand composite build up. He found 1 year success in one case, while 6 months for the other at the time of reporting of the cases. Wijlen has pointed out a few advantages of this technique First, as the procedure can be completed in one appointment, it has better patient compliance and reduces the cost of the appointment to the patient. Second, interdental spaces may be shaped to facilitate access for oral hygiene. Third, repairs can be carried out directly, without the need for any complicated techniques or materials. Fourth, control of the entire procedure remains with the operating dentist. Adjustments to the design, esthetic details and occlusal and soft-tissue relationships may be carried out immediately or in a minimum time during follow-up appointments.^8^

The technique employed in this procedure is very simple and the results are very esthetic and functionally sat isfactor y. More clinical research is required with larger number of samples to assess the long-term success of the appliance.

## CONCLUSION

Loss of an anterior tooth at younger age may result in psychological trauma to the child. The problem may become serious and deep seated and may result in an imbalanced emotional development of the child. Early loss of a deciduous tooth may also result in drifting of adjacent teeth leading to space loss. Thus, to address the functional and esthetic problem associated with the loss of an anterior tooth, the present appliance was placed using a simple technique with an esthetic and functionally satisfactory result.
